# Elemental Markers in Elasmobranchs: Effects of Environmental History and Growth on Vertebral Chemistry

**DOI:** 10.1371/journal.pone.0062423

**Published:** 2013-10-01

**Authors:** Wade D. Smith, Jessica A. Miller, Selina S. Heppell

**Affiliations:** 1 Department of Fisheries and Wildlife, Hatfield Marine Science Center, Oregon State University, Newport, Oregon, United States of America; 2 Department of Fisheries and Wildlife, Coastal Oregon Marine Experiment Station, Hatfield Marine Science Center, Oregon State University, Newport, Oregon, United States of America; 3 Department of Fisheries and Wildlife, Oregon State University, Corvallis, Oregon, United States of America; Biodiversity Insitute of Ontario - University of Guelph, Canada

## Abstract

Differences in the chemical composition of calcified skeletal structures (e.g. shells, otoliths) have proven useful for reconstructing the environmental history of many marine species. However, the extent to which ambient environmental conditions can be inferred from the elemental signatures within the vertebrae of elasmobranchs (sharks, skates, rays) has not been evaluated. To assess the relationship between water and vertebral elemental composition, we conducted two laboratory studies using round stingrays, *Urobatis halleri*, as a model species. First, we examined the effects of temperature (16°, 18°, 24°C) on vertebral elemental incorporation (Li/Ca, Mg/Ca, Mn/Ca, Zn/Ca, Sr/Ca, Ba/Ca). Second, we tested the relationship between water and subsequent vertebral elemental composition by manipulating dissolved barium concentrations (1x, 3x, 6x). We also evaluated the influence of natural variation in growth rate on elemental incorporation for both experiments. Finally, we examined the accuracy of classifying individuals to known environmental histories (temperature and barium treatments) using vertebral elemental composition. Temperature had strong, negative effects on the uptake of magnesium (D_Mg_) and barium (D_Ba_) and positively influenced manganese (D_Mn_) incorporation. Temperature-dependent responses were not observed for lithium and strontium. Vertebral Ba/Ca was positively correlated with ambient Ba/Ca. Partition coefficients (D_Ba_) revealed increased discrimination of barium in response to increased dissolved barium concentrations. There were no significant relationships between elemental incorporation and somatic growth or vertebral precipitation rates for any elements except Zn. Relationships between somatic growth rate and D_Zn_ were, however, inconsistent and inconclusive. Variation in the vertebral elemental signatures of *U. halleri* reliably distinguished individual rays from each treatment based on temperature (85%) and Ba exposure (96%) history. These results support the assumption that vertebral elemental composition reflects the environmental conditions during deposition and validates the use of vertebral elemental signatures as natural markers in an elasmobranch. Vertebral elemental analysis is a promising tool for the study of elasmobranch population structure, movement, and habitat use.

## Introduction

The trace and minor elemental composition of biomineralized structures can provide insight into the environmental conditions in which the elements were deposited. Elemental assays of coral skeletons and foraminifera tests, for example, have been commonly applied as surrogates of past climatic or oceanographic conditions (paleoproxies) [1–3]. Recently, considerable attention has been directed toward analyses of calcified structures, such as fish otoliths, to gain insight into contemporary ecological processes and inform management and conservation efforts [4–6]. Elements are naturally acquired through respiratory and dietary pathways and assimilated into actively calcifying structures such as scales, shells, and otoliths [7,8]. The elemental composition of these structures can reflect the physical and chemical conditions of the ambient environment. If the calcified material is deposited in a temporally consistent pattern and is not subjected to resorption or reworking, elemental composition can provide permanent chronological records of the environmental conditions experienced over a lifetime.

The most widespread and expanding application of elemental markers in biomineralized structures has occurred using the otoliths of fishes [4,5]. Otoliths are metabolically inert calcium carbonate structures (typically in the form of aragonite) that are used for balance and hearing in teleost fishes. Elements are incorporated into otoliths daily as new aragonite is crystallized onto an organic framework of proteins [8]. The elemental composition of other calcified structures, including vertebrae [9], scales [10], fin rays [11], and bone [12], have been evaluated as potential elemental markers in fishes. However, unlike otoliths, these calcium phosphate structures (in forms of hydroxyapatite) are metabolically active, subject to resorption and provide short-term and unstable chemical records of environmental history [4].

The elemental composition of biogenic calcified structures is not a simple reflection of environmental conditions. A variety of physiological barriers and processes are encountered as elements are taken up from the water through the gills or intestine, transferred through the blood plasma, and eventually incorporated into biomineralized structures [8]. Physiological regulation of internal elemental composition can result in active discrimination or preferential uptake of elements, thus modifying relationships with ambient environmental conditions. Trace metals such as manganese and zinc that are essential for metabolic and cellular transport processes are tightly regulated [13]. Conversely, physiological regulation of elements that do not play critical biological roles or generate toxic effects may be comparatively minimal [8,14]. At the site of calcification, elemental incorporation can be inhibited or promoted by kinetic effects associated with biomineralization. Elemental composition can be further modified by temperature, which has a profound influence on the rates of chemical and metabolic processes [15,16]. Individual variation in growth rates, independent of temperature, can also influence elemental composition [17,18]. Metabolic and kinetic effects on elemental incorporation, however, do not negate the utility of an element as a geochemical marker, providing the degree of regulation is constant or predictable.

Sharks, skates, and rays (elasmobranchs) are cartilaginous fishes that lack otoliths. Elasmobranch skeletons are composed of mineralized cartilage, an impure (non-stochiometric) form of carbonated calcium phosphate (hydroxyapatite) [19]. Like the otoliths of teleost fishes, elasmobranch vertebrae are deposited by the precipitation of elements onto a matrix of proteins and continue to grow throughout the life of the organism [19]. Vertebral growth bands are typically deposited seasonally, allowing individual ages to be determined. Resorption or physiological reworking of vertebrae, as has been observed in scale and bone hydroxyapatite, would alter the elemental composition and severely limit their utility as records of the physiochemical environment. Questions have been raised about the potential for elemental resorption in elasmobranch vertebrae [20], but directed studies have consistently found these structures to be stable [19,21,22]. The function and properties of elasmobranch cartilage, and vertebrae in particular, are fundamentally different than those of other vertebrates [21,23]. Whereas calcified cartilage is usually a transitional tissue that is ultimately replaced by bone, elasmobranch cartilage possesses a permanent mineralized rind that shows no direct evidence of remodeling or resorption [19,24]. Doyle [25] confirmed that mineralization and growth of elasmobranch cartilage is accomplished through surface accretion that proceeds without altering the mineral or protein matrix. Therefore, the elemental composition of elasmobranch vertebrae is unlikely to be modified after deposition and could therefore provide permanent chronological records of the environmental conditions experienced by individuals.

Chemical analyses of elasmobranch vertebrae to date have been predominately directed toward age validation [26,27] and dietary studies [28,29]. The potential use of vertebral elemental composition to delineate elasmobranch populations was first proposed by Edmonds et al. [30] following their analyses of jaw cartilage which revealed spatially explicit patterns of elemental variation. Age-related changes in vertebral elemental composition have recently been examined to discern movement patterns in sharks [31,32]. Although significant temporal and spatial variation in elemental composition have been identified within elasmobranch vertebrae, interpretations of these differences are hindered by a lack of understanding as to how ambient vertebral chemistry relates to environmental conditions. Without an understanding of the factors that influence elemental incorporation and the extent of regulation, it is impossible to know if the elemental composition of a calcified structure presents a reliable record of environmental history. Controlled laboratory validation studies provide a platform for quantifying abiotic and biotic effects on elemental incorporation and identifying those elements that are most likely to serve as useful indicators of the environment in which they were deposited. Incorrect assumptions about elemental relationships and an inadequate understanding of the mechanisms determining incorporation can lead to erroneous interpretations of field data.

Key assumptions regarding vertebral elemental incorporation in relation to the physical and chemical environment must be evaluated before broader ecological questions and hypotheses can be addressed using naturally occurring elemental markers in elasmobranchs. We quantified the effects of temperature and growth rate on vertebral elemental incorporation through controlled laboratory studies using the round stingray, *Urobatis halleri*, as a model species. We manipulated environmental concentrations of barium (Ba) to determine the extent to which vertebral elemental ratios reflect the ambient environment. Finally, we evaluated the utility of these elemental markers to distinguish the environmental history experienced by individual rays using multivariate classification models. These experiments allowed us to test the following hypotheses: (i) elemental incorporation in vertebrae is mediated by water temperature; (ii) vertebral Ba to calcium ratios (Ba/Ca) reflects water Ba/Ca; (iii) growth rate does not significantly influence vertebral elemental composition; and (iv) vertebral elemental markers can distinguish individuals based on differences in environmental history. This investigation represents the first attempt to evaluate the utility of vertebral chemistry as potential records of environmental history in elasmobranchs.

## Materials and Methods

### Ethics statement

This investigation was conducted with a permit from the California Department of Fish and Game (803099-01) and in strict accordance with guidelines established by the American Fisheries Society and National Institutes of Health for the use of fishes in research. Experimental protocol was approved by Oregon State University’s Institutional Animal Care and Use Committee (3783).

### Specimen collection

The round stingray, *Urobatis halleri*, is a benthic, live-bearing elasmobranch that occurs in estuaries and nearshore coastal soft bottom habitats from Panama to Eureka, California, USA [33]. The vertebrae of round rays are well-calcified and the annual deposition of a distinctive band pair (one opaque, one translucent growth band) has been validated, making reliable estimates of age and growth rates possible [34]. We selected *U. halleri* as a model elasmobranch species for vertebral elemental incorporation studies because of their record of hardiness in captivity, relatively small body size (to 31 cm disc width, DW), availability in shallow coastal environments, and validated periodicity of vertebral growth band formation.

Juvenile *U. halleri* were collected by beach seine at Seal Beach, California (33°44’ N; 118°06’ W) on 6 February 2009 and transported to the Hatfield Marine Science Center (HMSC) in Newport, Oregon. A total of 108 rays were collected, consisting of 67 females and 41 males. Vertebral band counts performed at the end of this study confirmed that these rays were age 0 (i.e. young-of-the-year; n = 104) and age 1 (n = 4) at the time of capture.

### Experimental design

We conducted two consecutive laboratory experiments using the same rays to evaluate the effects of: 1) temperature and 2) dissolved barium concentration on the incorporation of elements into the vertebrae of an elasmobranch. Following collection and transport, *U. halleri* were allowed to acclimate to lab conditions for five weeks. Total weight, DW, and sex were then recorded. Twelve rays were randomly assigned to each of nine 1,700 L independently re-circulating tanks containing a thin layer of sand substrate. Water from each tank circulated through individual wet-dry sumps containing biological filter media to reduce the build-up of potentially harmful nitrogenous waste products. The same combination of squid (*Doryteuthis opalescens*), herring (*Clupea pallasi*), or shrimp (*Pandalus jordani*) was provided daily. Remaining food and waste were removed daily. One-quarter to one-half volume water changes were completed approximately every 1-2 weeks to maintain water quality. Seawater was pumped from Yaquina Bay through HMSC’s seawater system. Tanks were covered with clear plastic lids to reduce evaporation. A 12 hour light:dark photoperiod was established for both experiments. Temperature and salinity were recorded daily and water samples were collected weekly.

### Temperature experiment

Following acclimation and initial measurement, all specimens were injected with a 25 mg kg^-1^ dose of oxytetracycline [35] to provide a visual indicator within the vertebrae that coincided with the start of the experiment. Cooler water from Yaquina Bay was heated and three replicate treatments of 15°C, 18°C, and 24°C were established and maintained, corresponding with the mean winter, summer, and approximate maximum water temperatures at the site of collection [36]. The temperature experiment was conducted for eight months (April-December, 2009) to ensure that adequate vertebral deposition occurred for elemental analysis in all treatments. Three individuals died before the conclusion of the study and were excluded from analysis; praziquantel (Sigma-Aldrich) was subsequently administered to all tanks (10 mg/L) to eliminate parasitic flatworms during two weeks in July and August.

### Barium manipulation experiment

The relationship between water chemistry and vertebral elemental composition was evaluated through experimental manipulation of dissolved barium concentrations. Barium was selected because of its utility as a distinctive elemental marker demonstrated in both field [37,38] and laboratory studies [39,40]. Upon conclusion of the temperature experiment, all specimens were weighed, measured, and injected with a 5 mg kg^-1^ dose of the fluorescent marker calcein to distinguish vertebral deposition between the experiments [41]. Water temperatures were gradually adjusted to 19°C in all tanks over two weeks. Following this acclimation, Ba treatments were systematically assigned to each tank to ensure that one tank from each prior temperature treatment was represented within each Ba treatment. Three tanks were designated as controls that reflected ambient dissolved Ba/Ca ratios (1x). Three tanks were spiked with three times (3x) and three tanks were spiked with six times (6x) the estimated mean local Ba concentration of 4.50 μmol mol^-1^, providing triplicate treatments of 1x, 3x, and 6x Ba concentrations. These values fall within the naturally occurring regional range for estuaries and coastal waters [40,42]. Elevated Ba treatments were prepared by the addition of BaCl_2_ (JT Baker) to ambient seawater. Diet, feeding, cleaning, and light regimes were maintained as previously described. Water changes were, however, made from an appropriate supply of 1x, 3x, or 6x Ba/Ca seawater sources. We excluded seven specimens from analysis (five from a single tank, 6x treatment) because of mortality that occurred prior to the completion of the study. All rays were sacrificed after 109 days (December, 2009 – April, 2010) using tricane methanesulfonate (Finquel, MS-222) in accordance with approved Institutional Animal Care and Use protocol. Rays were weighed and measured before vertebrae were excised and stored frozen for analysis.

### Vertebral preparation and elemental analysis

**Figure 1:**
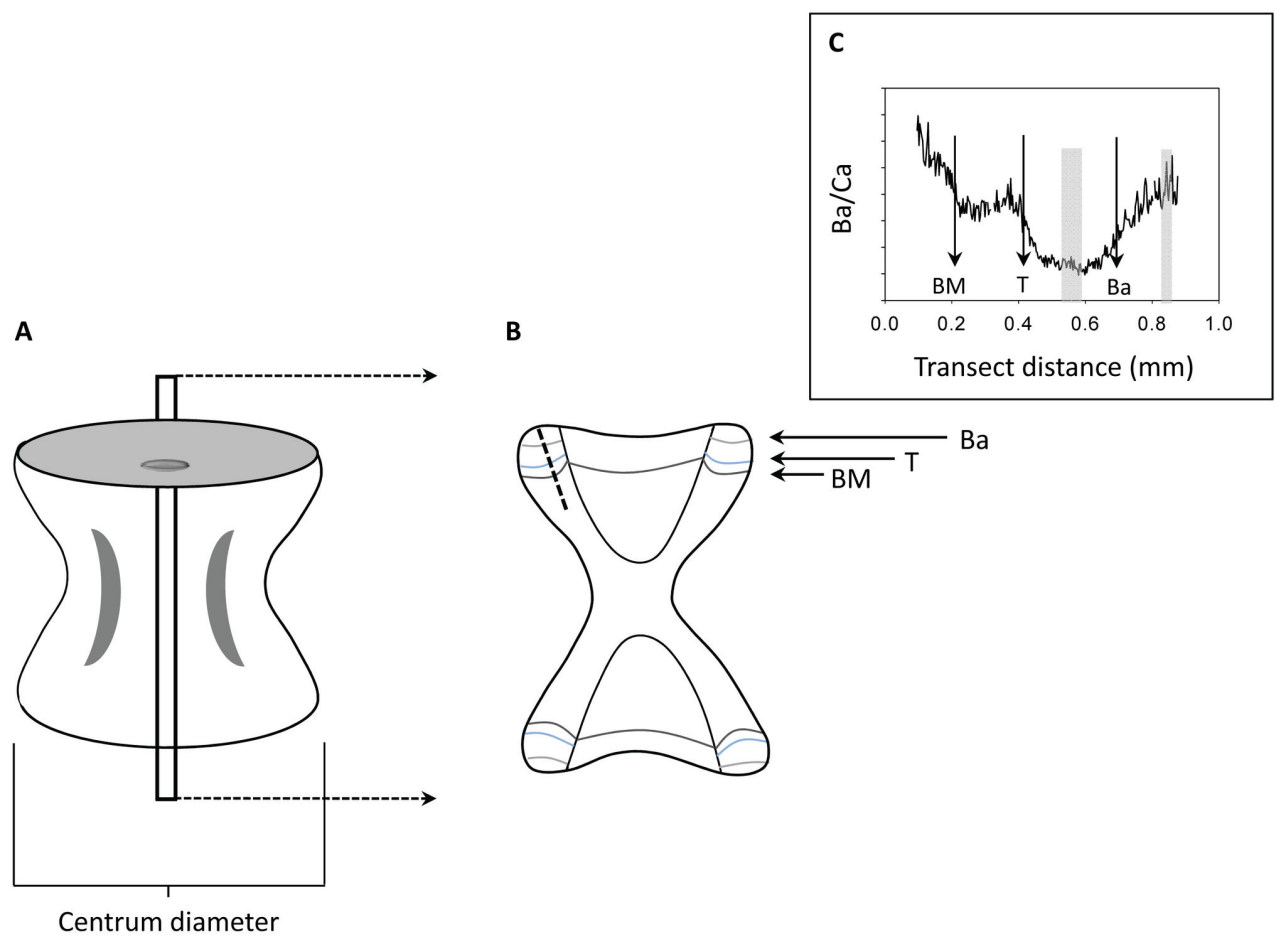
Processing and analysis of vertebral centra. Depiction of a (A) whole and (B) thin-sectioned vertebral centra and (C) the barium to calcium ratio (Ba/Ca) profile collected along a representative laser transect. The birthmark (BM) provides an intrinsic reference and fluorescent markers injected into round rays (Urobatis halleri) at the beginning of the temperature and barium manipulation experiments provided visual references for identifying specific regions deposited during this study. T represents the beginning of the temperature experiment (indicated by an oxytetracycline mark). Ba represents the beginning of the barium manipulation experiment which was distinguishable by a corresponding calcein mark. The dashed line in (B) exemplifies the transect pathway used for laser ablation. Grey vertical bars within (C) depict the regions integrated for analysis. The example in (C) characterizes the variation in vertebral Ba/Ca of a round ray that was maintained at 18°C and 6x ambient Ba concentration during the temperature and barium manipulation experiments, respectively.

Sample preparation for elemental analysis followed procedures typical to age and growth studies of elasmobranchs [43] but incorporated processing methods associated with otolith chemistry studies [44] to minimize contamination. Tissue was removed from vertebrae with acid-washed non-metallic dissecting tools and individual centra were separated and dried in a Class 100 laminar flow bench. Vertebral centra were soaked for 10 minutes in ultrapure 30% hydrogen peroxide (ULTREX, J.T. Baker) to loosen remaining connective tissue, triple rinsed, and ultrasonically cleaned in Nanopure® (18 M Ohm, Barnstead International) water for 45 minutes. Samples were rinsed, dried, embedded in polyester casting resin infused with a spike of indium, and sectioned to a width of ~0.4 mm using a low speed diamond saw (Figure 1a, 1b). Resulting thin-sections were mounted to acid-washed glass slides, polished with lapping film (3M^TM^; 30, 12, 5, 3, 1 μm), and rinsed. Sectioned centra were randomly attached to acid-washed slides to prevent systematic bias. Sample slides were rinsed with ultrapure 1% nitric acid (HNO_3_; ULTREX, J.T. Baker), cleaned ultrasonically for 15 minutes, triple rinsed, and dried in Class 100 conditions.

The elemental composition of *U. halleri* vertebrae was quantified using laser ablation-inductively coupled plasma mass spectrometry (LA-ICPMS). Analyses were conducted at Oregon State University’s WM Keck Collaboratory for Plasma Spectrometry in Corvallis, Oregon using a VG PQ ExCell ICPMS with a DUV193 excimer laser (New Wave Research). The laser was set at a pulse rate of 5 Hz with an ablation spot size of 80 μm and translated across the sample at 5 μm s^-1^. Laser transects were positioned within the corpus calcareum of the vertebral centrum to collect a time series of elemental composition that included both experimental periods (Figure 1b). Transects were pre-ablated (100μm spot size, 2 Hz, 100 μm s^-1^) to further reduce potential sample contamination. We collected data on 17 elements: lithium, magnesium, calcium, titanium, vanadium, chromium, manganese, cobalt, copper, zinc, rubidium, strontium, zirconium, cadmium, barium, lanthanum, and lead. However, only magnesium (^25^Mg), calcium (^43^Ca), manganese (^55^Mn), zinc (^66^Zn), strontium (^88^Sr), and barium (^138^Ba) were consistently above detection limits. Lithium (^7^Li) was often found in concentrations near and occasionally below detection limits but was included in our analyses. Samples with measurements of Li that were not above background levels were dropped from analysis (19% temperature experiment, 29% Ba manipulation experiment). Lead was incorporated into vertebrae at levels exceeding detection limits when specimens were living off Seal Beach, CA. However, Pb/Ca ratios were not consistently above detection limits (≥4.19 μmol mol^-1^) while rays were maintained at HMSC.

Data processing followed procedures described in Miller & Shanks [44]. To evaluate instrument drift and daily variation in instrument sensitivity, a National Institute of Standards and Technology (NIST) 612 glass standard was run with each sample slide. Background levels of analyte isotopes were measured and subtracted from values determined during vertebral ablation. Mean percent relative standard deviations (%RSD) of the NIST 612 standard were: Li = 5.2%, Mg = 12.6%, Ca = 3.3%, Mn = 4.5%, Zn = 8.3%, Sr = 3.7%, and Ba = 5.0%(n = 21). Time-resolved software (PlasmaLab®) allowed analyte counts to be integrated from specific positions along each vertebral transect. Regions for integration were determined using image analysis (Image ProExpress, Media Cybernetics®). We targeted areas that corresponded with the mid-point of the temperature experiment and the final month of the Ba manipulation experiment for analysis to assure that adequate vertebral precipitation had occurred and to avoid sampling areas associated with the transition between experiments (Figure 1c). Count data were normalized by ^43^Ca to adjust for variability in instrument sensitivity and the amount of ablated material, then converted to elemental ratios (Me/Ca, where Me represents a metallic element) based on measurements of the NIST 612 standard [45,46]. Elemental ratios are presented in mmol mol^-1^ (Mg, Sr) or μmol mol^-1^ (Li, Mn, Zn, Ba).

### Water collection and analysis

Dissolved elemental concentrations within and among treatments were evaluated by sampling the water from each tank weekly over the course of both experiments. Samples were collected in acid-washed plastic bottles, filtered with 0.2-μm syringe filters in a Class 100 laminar flow bench, acidified to <2 pH with ultrapure HNO_3_ (ULTREX, J.T. Baker) and stored refrigerated at ~4°C until analysis. A subset of samples was analyzed to determine the concentrations of Li, Mg, Ca, Mn, Zn, Sr, and Ba during the temperature (n = 11 dates x 9 tanks) and Ba manipulation (n = 7 dates x 9 tanks) experiments. Water samples were selected to provide increased representation during the middle of the temperature experiment (August-October) and the latter half of the Ba manipulation experiment (February-March), the same time period from which vertebral elemental data were targeted.

Elemental concentrations were determined using a Leeman-Teledyne inductively coupled plasma optical emission spectrometer (ICP-OES) (Li at 670.8 nm, Mg at 279.1 nm, Ca at 317.9 nm, Mn at 259.4 nm, Zn at 206.2, Sr at 421.5 nm, and Ba at 493.4 nm). Filtered, acidified samples were diluted 100x for the determination of Mg, Ca, and Sr and 25x for Li, Mn, Zn, and Ba. Matrix-matched standards were created using SPEX Certiprep Group® certified reference materials (CRMs), NIST liquid standard (1643e), and a sodium chloride (NaCl) solution. Matrix-matched NIST standards and HNO_3_ blanks were introduced throughout analysis to evaluate accuracy. Measured Li, Mg, Ca, Mn, Zn, Sr, and Ba concentrations were within 3%, 2%, 3%, 18%, 8%, 5%, and 3%, respectively, of certified values. A correction factor was applied to those elements that were ≥5% of known values (Mn, Zn and Sr). Repeated measurements of the same CRM calibration standard indicated that precision was within 1.2% for all elements (n = 7). Elemental concentrations are expressed as element to calcium ratios and presented in mmol mol^-1^ (Mg, Sr) or μmol mol^-1^ (Li, Mn, Zn, Ba).

### Statistical analyses

Partition coefficients (D_Me_, where the subscript indicates a metallic element of interest) characterize the relationship between the elemental composition of a solution with that of a solid, actively calcifying structure [47]. D_Me_ provide a standardized metric for comparing the effects of temperature, dissolved elemental concentration, and growth rates on elemental incorporation within and among species and calcified structures. We calculated D_Me_ for each element by dividing a given element to calcium ratio (Me/Ca) measured from individual vertebrae by the mean Me/Ca ratio measured from the water of the corresponding tank [47].

Mean salinity, temperature, Me/Ca_water_, and Me/Ca_vertebrae_ were compared among treatments using parametric and non-parametric approaches. Data were screened for outliers and assessed for normality and homogeneity of variance using Shapiro-Wilk’s and Levene’s tests, respectively [48]. Temperature and salinity data did not meet the assumptions of normality following transformation and were analyzed using non-parametric Kruskal-Wallis analysis of variance by ranks [48]. Water (Me/Ca_water_) and vertebral elemental (Me/Ca_vertebrae_, D_Me_) data required log_10_-transformation to conform to the assumptions of parametric statistical analysis.

Data collected from the temperature and Ba manipulation experiments were analyzed separately using the same procedures. As a first step, one-way multivariate analysis of variance (MANOVA) was applied to test for differences in mean Me/Ca_water_ and Me/Ca_vertebrae_ among treatments, where treatment (temperature or Ba concentration) was a fixed factor and elemental ratios (Li/Ca, Mg/Ca, Mn/Ca, Zn/Ca, Sr/Ca, Ba/Ca) were the response variables. When significant differences among treatments were identified, Tukey’s Honestly Significant Difference (THSD) tests were conducted to determine which groups accounted for the observed differences [49]. Effects of temperature and Ba concentration on D_Me_ were evaluated in each experiment by ANOVA with tanks nested within treatments as random variables and the corresponding temperature or Ba treatment as a fixed factor. All MANOVAs and nested ANOVAs were completed using JMP (Version 8.0) statistical software.

### Effects of growth and precipitation rates

Somatic growth and vertebral precipitation rates were determined to evaluate their influence on elemental incorporation. Because rays were not individually marked, we assumed that sex-specific size ranks were maintained within each tank during the experiments and estimated individual growth rates from these ranks [50]. Somatic growth rates were calculated as the difference in body size (DW) between the start and end of each experiment divided by the number of months that the experiment was conducted, providing an estimate of growth in mm DW month^-1^. Changes in centrum diameter during each experiment were similarly calculated by subtracting the vertebral diameter at the beginning of a study, as indicated by a fluorescent mark, from that measured at the end of an experiment using Image Pro Plus® (Media Cybernetics). Vertebral deposition/precipitation rates were expressed as mm DW month^-1^. Mean monthly growth rates were estimated for all tanks (n = 9) and compared among treatments using ANOVA with treatment as a fixed factor. Regression analyses of D_Me_ against somatic growth and vertebral precipitation rates were performed within each treatment.

### Classification

The ability to accurately classify individuals based on treatment/environmental history was evaluated with discriminant function analysis (DFA) of the vertebral Me/Ca data (i.e. Mg, Mn, Sr, Ba) generated from our temperature and Ba manipulation experiments. Because Li/Ca measurements were not available for all samples (i.e. below detection limits), Li was not included in these analyses. Group classification accuracy was assessed using a leave-one-out jack-knife procedure [51]. We assumed that prior probabilities of group membership were proportional to group sample sizes. A chance-corrected classification (Cohen’s kappa, κ) was also calculated to determine if predicted group assignments exceeded that of randomly assigning individuals to groups in proportion to their sample sizes [52]. A κ of 0 indicates that no improvement over chance was provided by the DFA and a κ of 1 signifies perfect agreement. SYSTAT (Version 12.0) was used for DFA.

## Results

### Temperature experiment

Water temperatures differed significantly among treatments, as intended (Kruskal-Wallis, H = 69.96, *p* < 0.001; Table 1). Salinity varied during the experiment but remained equivalent among and within treatments (Kruskal-Wallis: H = 4.79, *p* = 0.09; Table 1). Additionally, water elemental ratios did not differ in response to temperature (MANOVA, Pillai’s trace = 0.15, *p* = 0.74; Table 2). Of the six elemental ratios measured, Zn/Ca_water_ displayed the greatest variation (overall %CV = 47.5) and Sr/Ca_water_ the least (overall %CV = 1.1).

**Table 1:** Mean experimental conditions by tank and treatment during the temperature experiment.

Tank	**Treament (°C)**	**n**	**Mean DW (mm)**	**Temp (°C)**	**Salinity**	**Li/Ca_water_ (μmol mol^-1^)**	**Mg/Ca_water_ (mmol mol^-1^)**	**Mn/Ca_water_ (μmol mol^-1^)**	**Zn/Ca_water_ (μmol mol^-1^)**	**Sr/Ca_water_ (mmol mol^-1^)**	**Ba/Ca_water_ (μmol mol^-1^)**
3	15	12	115.8 (17.4)	15.4 (1.2)	31.6 (1.6)	11.57 (0.26)	4988 (20.8)	13.64 (0.76)	18.88 (7.91)	8.77 (0.09)	6.31 (1.52)
6	15	12	117.5 (16.2)	15.0 (1.2)	31.6 (1.6)	11.54 (0.34)	4963 (61.9)	13.45 (0.52)	19.44 (9.55)	8.74 (0.12)	5.35 (1.48)
8	15	11	118.0 (20.5)	15.5 (1.3)	31.7 (1.6)	11.43 (0.34)	4984 (15.3)	13.45 (0.28)	27.42 (17.20)	8.76 (0.09)	5.14 (1.65)
1	18	11	100.0 (18.0)	18.8 (0.9)	32.3 (1.2)	11.63 (0.27)	4992 (18.8)	13.50 (0.24)	24.37 (10.57)	8.79 (0.07)	5.05 (0.95)
4	18	10	121.3 (20.8)	18.5 (0.8)	32.2 (1.2)	11.70 (0.21)	4990 (12.3)	13.76 (0.30)	20.51 (7.78)	8.79 (0.07)	5.22 (1.07)
7	18	12	115.9 (16.6)	18.6 (0.7)	32.4 (1.2)	11.64 (0.47)	4999 (18.5)	13.64 (0.54)	22.23 (13.27)	8.81 (0.08)	5.39 (1.24)
2	24	12	116.4 (19.6)	23.9 (0.9)	32.6 (1.4)	11.43 (0.64)	4984 (31.7)	14.26 (1.08)	24.10 (6.13)	8.78 (0.14)	5.37 (1.52)
5	24	12	119.7 (18.3)	24.3 (1.2)	33.0 (1.9)	11.42 (0.54)	4987 (17.4)	13.40 (1.28)	24.85 (13.90)	8.78 (0.11)	5.02 (1.30)
9	24	13	115.6 (14.2)	24.1 (0.8)	33.2 (1.4)	11.44 (0.33)	4983 (13.1)	13.68 (0.79)	28.18 (10.71)	8.76 (0.08)	5.63 (1.28)

The summary includes the number of round rays (*Urobatis halleri*) per tank (n, their mean size in disc width (DW) at the onset of the study, water temperature (°C), salinity, and dissolved element to calcium (Ca) ratios for lithium (Li), magnesium (Mg), manganese (Mn), zinc (Zn), strontium (Sr), and barium (Ba). Values in parenthesis are ± standard deviation. Untransformed element to calcium ratios are presented below but log_10_ transformation was necessary to meet the assumptions for parametric statistical analysis.

**Table 2:** Univariate results from multivariate analysis of variance tests to evaluate the effect of temperature (Temp; 15°C, 18°C, and 24°C) on dissolved element to calcium ratios (Me/Ca) in water and vertebral Me/Ca among treatments.

**Source**	**Me/Ca**	**Effect**	**DF**	**MSE**	**F**	**p**
Water	Li	Temp	2	< 0.001	3.32	0.107
		(Tank)	6	< 0.001		
	Mg	Temp	2	< 0.001	1.76	0.250
		(Tank)	6	< 0.001		
	Mn	Temp	2	< 0.001	0.27	0.774
		(Tank)	6	0.001		
	Zn	Temp	2	0.005	1.71	0.258
		(Tank)	6	0.003		
	SrSr	Temp	2	< 0.001	2.98	0.126
		(Tank)	6	< 0.001		
	Ba	Temp	2	0.001	0.46	0.652
		(Tank)	6	0.001		
Vertebrae	Li	Temp	2	0.015	1.56	0.284
		(Tank)	6	0.010		
	Mg	Temp	2	0.002	49.81	**< 0.001**
		(Tank)	6	< 0.001		
	Mn	Temp	2	0.019	8.87	**0.016**
		(Tank)	6	0.002		
	Zn	Temp	2	0.028	12.97	**0.007**
		(Tank)	6	0.002		
	SrSr	Temp	2	< 0.001	3.15	0.102
		(Tank)	6	< 0.001		
	Ba	Temp	2	0.118	91.78	**< 0.001**
		(Tank)	6	0.001		

Significant p-values are indicated by bold font. Data were log_10_-transformed prior to analysis.

**Figure 2:**
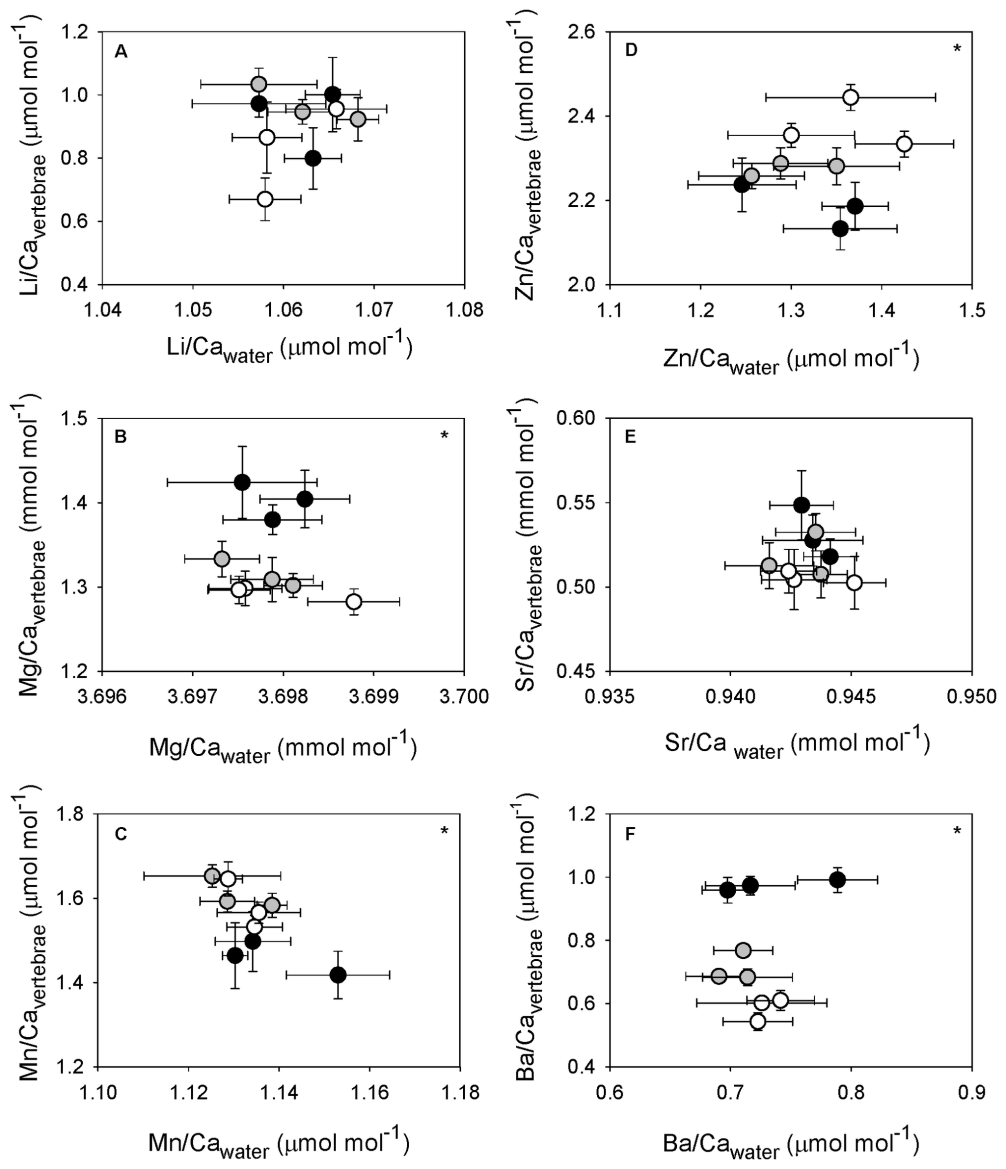
Relationships between water and vertebral elemental ratios by temperature treatment. Mean ± standard error of element to calcium ratios for water and vertebral samples. Black circles represent 15°C treatments, grey circles 18°C treatments, and open circles 24°C treatments. (A) lithium, (B) magnesium, (C) manganese, (D) zinc, (E) strontium, and (F) barium. Element to calcium ratios were log_10_-transformed. Significant temperature effects are indicated by (*).

We observed significant and varied responses in vertebral elemental composition among temperature treatments (MANOVA, Pillai’s trace = 10.80, *p* < 0.001; Table 2). Vertebral Li/Ca and Sr/Ca did not vary among temperatures (Figure 2a, 2e). Vertebral incorporation of Mg/Ca and Ba/Ca was significantly and negatively affected by temperature (Figure 2b, 2f) whereas incorporation of Mn/Ca and Zn/Ca was significantly and positively related to temperature (Figure 2c, 2d). The significant effect of temperature on both Mg/Ca_vertebrae_ and Mn/Ca_vertebrae_ was attributed to differences in the lowest temperature treatment. Mg/Ca_vertebrae_ was elevated at 15°C (THSD, *p* < 0.001 for 15° v. 18°C and 15° v. 24°C), however, Mn/Ca_vertebrae_ at 15°C was significantly less than those measured in *U. halleri* maintained 18° and 24°C (THSD, *p* = 0.009 for 15° v. 18°C and *p* = 0.005 for 15° v. 24°C). Mean Zn/Ca_vertebrae_ was significantly greater at 24°C but did not differ between 15 and 18°C treatments (THSD, *p* < 0.001 for 15 v. 24°C, *p* = 0.019 for 18 v. 24°C). Significant variation in Ba/Ca_vertebrae_ was evident across all treatments, with mean Ba/Ca_vertebrae_ decreasing with increasing temperature (Figure 2f; THSD, *p* < 0.001 for all pair-wise comparisons). Overall mean (± standard deviation, SD) Ba/Ca_vertebrae_ was 0.97 ± 0.12, 0.71 ± 0.08, and 0.59 ± 0.09 μmol mol^-1^ for 15°, 18°, and 24°C treatments, respectively.

**Figure 3:**
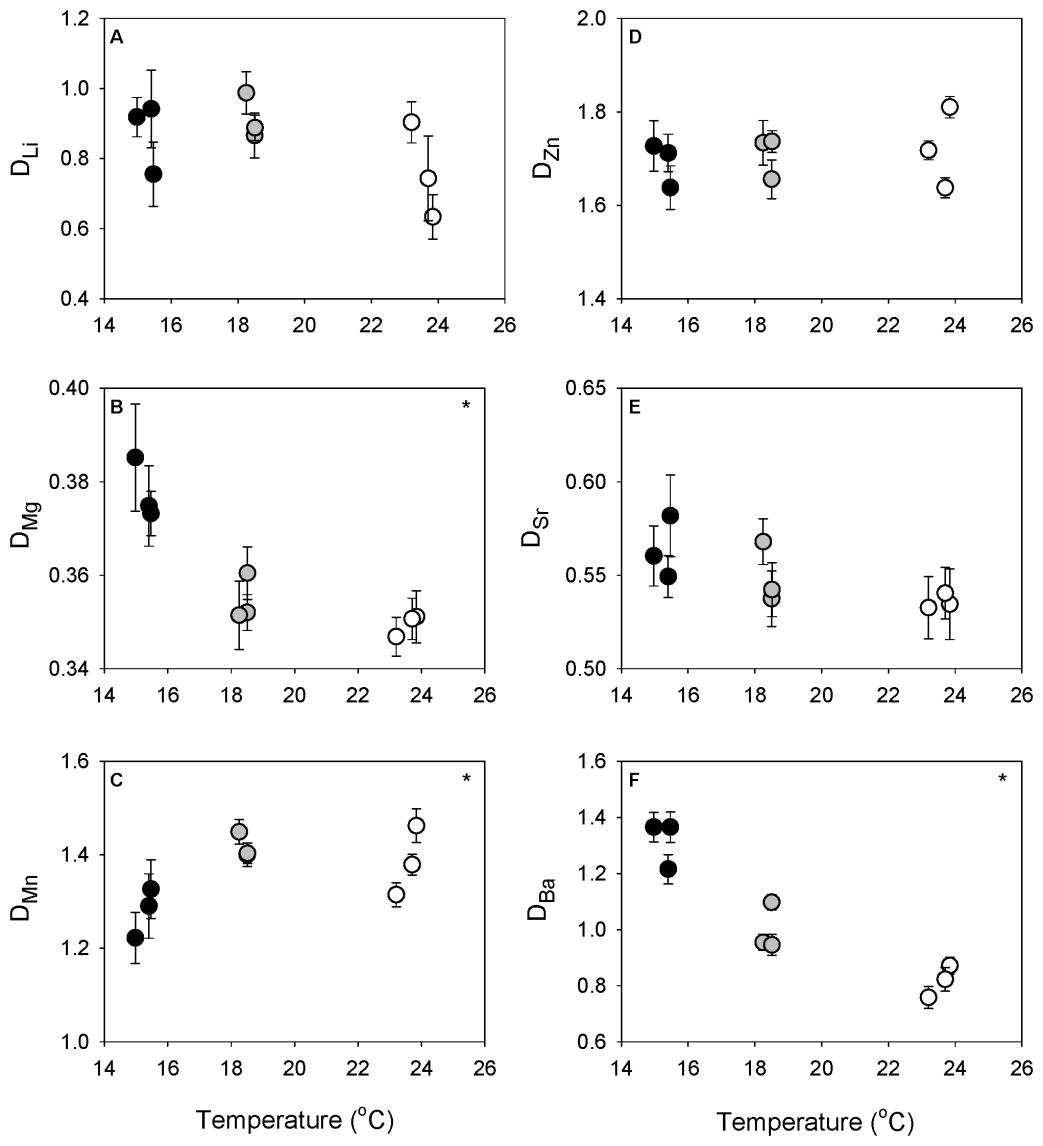
Influence of temperature treatment on elemental partitioning. Mean ± standard error of partition coefficients (D_Me_) for (A) lithium, (B) magnesium, (C) manganese, (D) zinc, (E) strontium, and (F) barium by treatment and mean tank temperature. Black circles represent 15°C treatments, grey circles 18°C treatments, and open circles 24°C treatments. Significant responses of D_Me_ to temperature are indicated by (*).

Varied responses to temperature were also observed among the partition coefficients calculated in this study (Table 3, Figure 3). We detected no effect of temperature on Li incorporation. Although a significant temperature effect was associated with Zn/Ca_vertebrae_, D_Zn_ indicated no evidence of temperature dependence. D_Sr_ values showed a slight decrease with increasing temperatures, but the observed trend was statistically insignificant. Temperature had a significant negative effect on D_Mg_ and D_Ba_ (Figure 3b, 3f) and positively influenced D_Mn_ (Figure 3c). Mean D_Mg_ declined with increasing temperature but the observed pattern was driven by differences between the 15°C and warmer treatments (THSD, *p* < 0.001 for 15° v. 18°C and 15° v. 24°C). A strong, negative effect of temperature on D_Ba_ was detected across treatments (THSD, *p* < 0.001 for all pair-wise comparisons). For D_Ba_, treatment means (± SD) were 1.31 ± 0.18, 0.99 ± 0.12, and 0.81 ± 0.13 at 15°, 18°, and 24°C, respectively. The positive effect of temperature demonstrated by D_Mn_ was due to increased discrimination of Mn at 15°C (lower D_Mn_ values) compared with 18° and 24°C (THSD, *p* = 0.017 for 15° v. 18°C and *p* = 0.001 for 15° v. 24°C).

**Table 3:** Results of nested analysis of variance to evaluate the effect of temperature (Temp; 15°C, 18°C, and 24°C) and barium ([Ba]; 1x, 3x, and 6x average ambient values) treatments on mean partition coefficients (D_Me_).

**Experiment**	**D_Me_**	**Effect**	**DF**	**MSE**	**F**	**p**
Temperature	D_Li_	Temp	2	0.017	2.32	0.180
		(Tank)	6	0.007		
	D_Mg_	Temp	2	0.001	47.86	**< 0.001**
		(Tank)	6	< 0.001		
	D_Mn_	Temp	2	0.016	5.90	**0.038**
		(Tank)	6	0.003		
	D_Zn_	Temp	2	< 0.001	0.17	0.850
		(Tank)	6	0.004		
	D_Sr_	Temp	2	< 0.001	3.09	0.119
		(Tank)	6	< 0.001		
	D_Ba_	Temp	2	0.191	31.77	**< 0.001**
		(Tank)	6	0.006		
Barium	D_Li_	[Ba]	2	0.003	1.50	0.297
manipulation		(Tank)	6	0.002		
	D_Mg_	[Ba]	2	< 0.001	1.69	0.261
		(Tank)	6	< 0.001		
	D_Mn_	[Ba]	2	0.002	1.96	0.221
		(Tank)	6	0.001		
	D_Zn_	[Ba]	2	0.137	0.61	0.575
		(Tank)	6	0.023		
	D_Sr_	[Ba]	2	< 0.001	0.27	0.769
		(Tank)	6	0.001		
	D_Ba_	[Ba]	2	0.015	20.44	**0.002**
		(Tank)	6	0.001		

Significant p-values are indicated by bold font. Data were log_10_-transformed prior to analysis.

### Ba manipulation experiment

**Figure 4:**
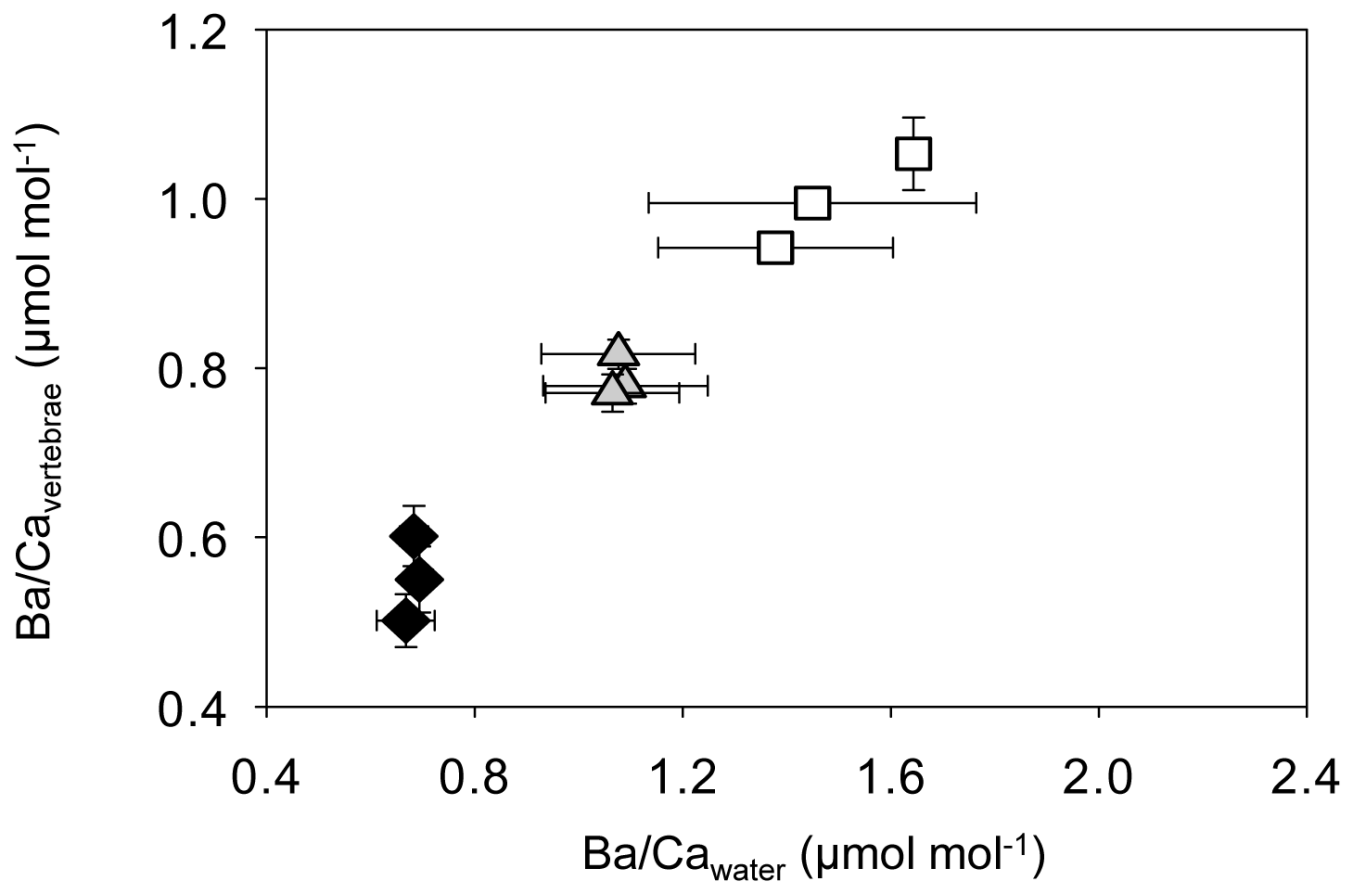
Relationships between water and vertebral barium to calcium ratios by barium treatment. Mean ± standard error of barium to calcium ratios for water and vertebral samples. Black diamonds represent 1x, grey triangles 3x, and unfilled squares 6x barium treatments. Element to calcium ratios were log_10_-transformed.

Targeted Ba concentrations of 3x and 6x were successfully attained (Tables 4, 5). Mean Ba/Ca_water_ values differed significantly among treatments (Figure 4; THSD, *p* < 0.01 for all pair-wise comparisons). Salinity (Kruskal-Wallis, H = 5.71, p = 0.06) and temperature (Kruskal-Wallis, H = 1.18, *p* = 0.56) did not differ among treatments (Table 4). With the exception of the intended variation in Ba/Ca_water_, dissolved elemental composition did not differ among treatments (MANOVA, Pillai’s trace = 0.72, *p* = 0.03; Table 5).

**Table 4:** Mean experimental conditions by tank and treatment during the barium manipulation experiment.

**Tank**	**Treament**	**n**	**Mean DW (mm)**	**Temp (°C)**	**Salinity**	**Li/Ca_water_ (μmol mol^-1^)**	**Mg/Ca_water_ (mmol mol^-1^)**	**Mn/Ca_water_ (μmol mol^-1^)**	**Zn/Ca_water_ (μmol mol^-1^)**	**Sr/Ca_water_ (mmol mol^-1^)**	**Ba/Ca_water_ (μmol mol^-1^)**
2	1x	12	162.1 (14.0)	19.8 (0.2)	30.0 (0.7)	11.59 (0.28)	4988 (8.8)	14.18 (0.74)	44.54 (13.22)	8.76 (0.05)	4.68 (0.52)
6	1x	12	131.8 (15.5)	19.6 (0.4)	30.7 (0.6)	11.87 (0.42)	4984 (11.0)	13.77 (0.57)	24.34 (6.56)	8.76 (0.07)	4.85 (0.60)
7	1x	12	143.5 (12.6)	19.5 (0.2)	29.9 (0.7)	12.06 (0.64)	5001 (32.9)	13.24 (0.67)	27.08 (9.37)	8.80 (0.12)	4.96 (0.50)
3	3x	11	132.3 (15.9)	19.8 (0.4)	29.5 (1.0)	11.74 (0.35)	4996 (26.1)	13.95 (0.78)	23.12 (4.72)	8.78 (0.13)	10.21 (7.83)
4	3x	10	146.6 (15.4)	19.5 (0.4)	29.8 (0.9)	11.73 (0.30)	4985 (16.1)	14.54 (1.28)	25.08 (11.05)	8.76 (0.09)	16.94 (14.11)
9	3x	12	165.5 (7.7)	19.4 (0.3)	29.8 (1.0)	11.55 (0.37)	4986 (16.7)	14.67 (0.56)	48.99 (20.95)	8.76 (0.07)	14.15 (8.30)
1	6x	11	148.2 (9.5)	19.4 (0.3)	29.8 (0.7)	11.89 (0.79)	5005 (39.1)	14.86 (1.37)	47.85 (22.22)	8.74 (0.07)	31.99 (21.58)
5	6x	7	167.4 (9.5)	19.8 (0.2)	29.7 (0.8)	11.81 (0.25)	4993 (18.9)	14.80 (1.91)	35.90 (21.38)	8.78 (0.11)	44.22 (3.87)
8	6x	11	136.5 (17.9)	19.7 (0.4)	29.8 (1.0)	11.73 (0.22)	4994 (34.2)	13.89 (0.59)	36.10 (15.18)	8.79 (0.12)	31.73 (19.23)

Treatments reflect ambient (1x) barium concentrations and targeted concentrations of three (3x) and six (6x) times the mean ambient value. The summary includes the number of round rays (*Urobatis halleri*) per tank (n), their mean size in disc width (DW) at the onset of the barium manipulation experiment, water temperature (°C), salinity, and dissolved element to calcium (Ca) for lithium (Li), magnesium (Mg), manganese (Mn), zinc (Zn), strontium (Sr), and barium (Ba). Values in parenthesis are ± standard deviation. Untransformed element to calcium ratios are presented below but log_10_-transformation of these data was necessary to meet the assumptions for parametric statistical analysis.

**Table 5:** Univariate results from multivariate analysis of variance tests to evaluate the effect of barium concentration ([Ba]; 1x, 3x, and 6x local ambient values) on dissolved element to calcium ratios in water and vertebral element to calcium ratios among treatments.

**Source**	**Me/Ca**	**Effect**	**DF**	**MSE**	**F**	**p**
Water	Li	[Ba]	2	< 0.001	1.49	0.299
		(Tank)	6	< 0.001		
	Mg	[Ba]	2	< 0.001	0.70	0.534
		(Tank)	6	< 0.001		
	Mn	[Ba]	2	0.001	5.08	0.051
		(Tank)	6	< 0.001		
	Zn	[Ba]	2	0.016	0.91	0.451
		(Tank)	6	0.017		
	SrSr	[Ba]	2	< 0.001	1.08	0.398
		(Tank)	6	< 0.001		
	Ba	[Ba]	2	0.424	28.19	**< 0.001**
		(Tank)	6	0.015		
Vertebrae	Li	[Ba]	2	0.004	1.14	0.382
		(Tank)	6	0.004		
	Mg	[Ba]	2	< 0.001	0.92	0.447
		(Tank)	6	< 0.001		
	Mn	[Ba]	2	< 0.001	0.16	0.858
		(Tank)	6	0.002		
	Zn	[Ba]	2	0.004	0.83	0.480
		(Tank)	6	0.002		
	SrSr	[Ba]	2	< 0.001	0.19	0.829
		(Tank)	6	< 0.001		
	Ba	[Ba]	2	0.145	99.59	**< 0.001**
		(Tank)	6	0.001		

Significant p-values are indicated by bold font. data were log_10_-transformed prior to analysis.

Ambient Ba concentration had a positive effect on Ba/Ca_vertebrae_ (MANOVA, Pillai’s trace = 0.95, *p* < 0.001; Table 5). Significant differences were found across treatments (Figure 4; THSD, *p* < 0.001 for all pair-wise comparisons). Mean (± SD) vertebral Ba/Ca were 0.56 ± 0.11, 0.76 ± 0.05, and 0.99 ± 0.09 μmol mol^-1^ for 1x, 3x, and 6x treatments, respectively.

**Figure 5:**
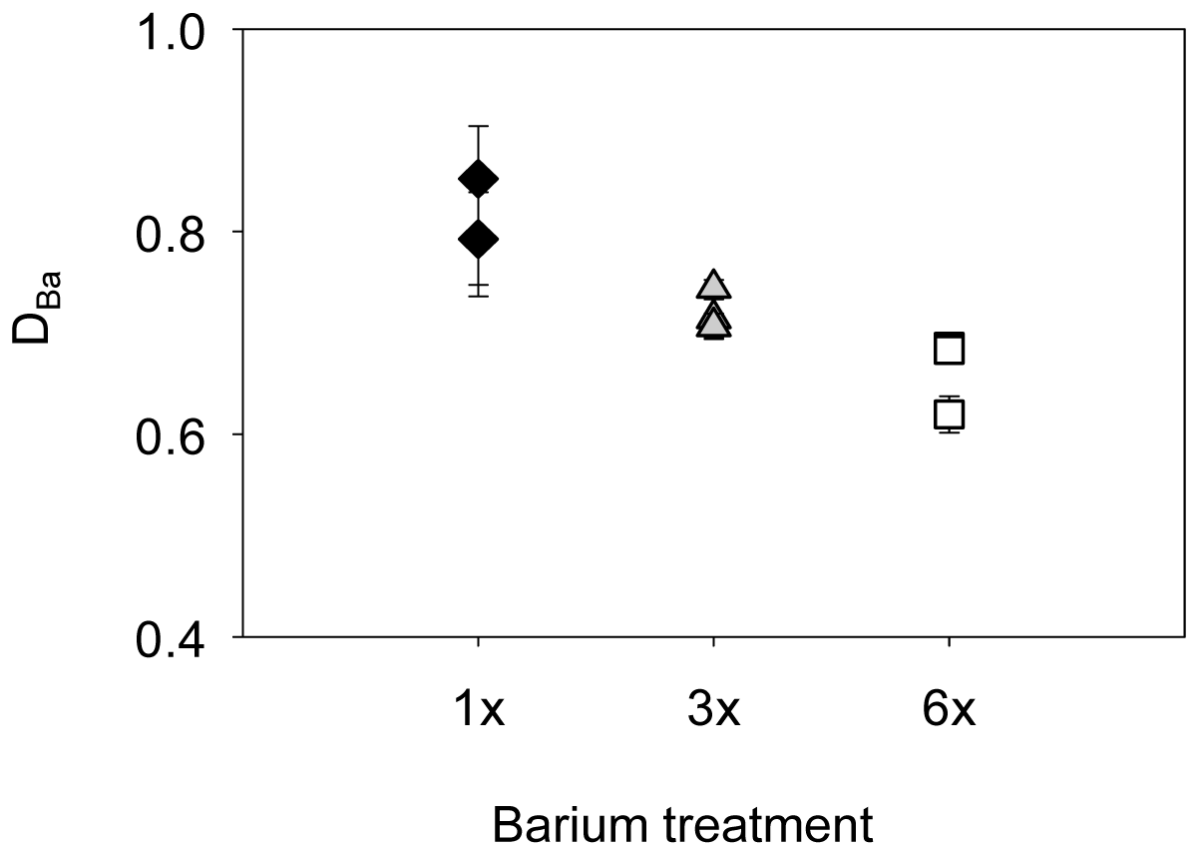
Influence of barium treatment on elemental partitioning. Mean ± standard error of barium partition coefficients (D_Ba_) by barium treatment. Black diamonds represent 1x, grey triangles 3x, and unfilled squares 6x that of average local barium values.

D_Ba_ decreased significantly with increasing dissolved Ba concentrations (Table 3, Figure 5). This negative relationship indicates that discrimination of Ba increases (less Ba is incorporated) in response to elevated environmental Ba concentrations. Mean D_Ba_ differed significantly among treatments (THSD, *p* < 0.01 for all pair-wise comparisons). Treatment means (± SD) of D_Ba_ were 0.81 ± 0.15 at 1x, 0.72 ± 0.05 at 3x, and 0.67 ± 0.05 at 6x.

### Precipitation and growth rate effects

**Figure 6:**
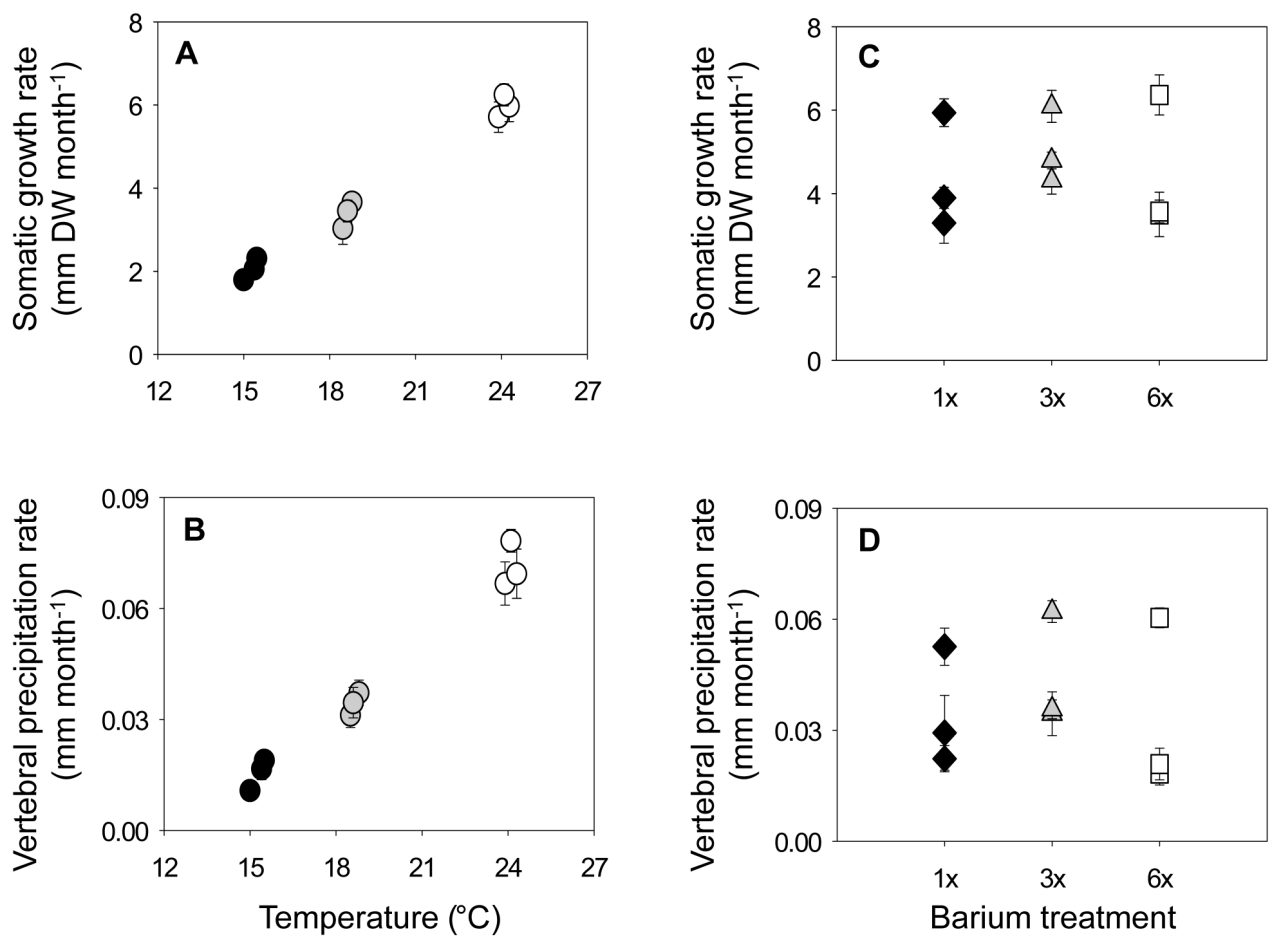
Mean somatic growth and vertebral precipitation rates during temperature and barium manipulation experiments. Mean (A, C) somatic growth and (B, D) vertebral precipitation rates by tank, treatment, and experiment (± standard error). (A, B) Black circles represent 15°C treatments, grey circles 18°C treatments, and open circles 24°C treatments. (C, D) Black diamonds represent 1x, grey triangles 3x, and unfilled squares 6x that of average local barium values. Temperatures were equivalent among treatments (19°C) during the barium manipulation experiment.

As anticipated, somatic growth (ANOVA, F_2,6_ = 148.40, *p* < 0.001) and vertebral precipitation (ANOVA, F_2,6_ = 115.53, < 0.001) rates were significantly affected by temperature. Mean growth rates increased with increasing temperatures, ranging between 1.8–6.2 mm DW month^-1^ (Figure 6a, Table S1). Vertebral deposition rates reflected a similar, positive response to temperature (Figure 6b). No significant relationships were identified between D_Me_ and somatic growth (*r* ≤ 0.30, *p* ≥ 0.10) or vertebral precipitation rates within temperature treatments (*r* ≤ 0.41, *p* ≥ 0.12) (Table S2), indicating that growth rates were not responsible for the variation in elemental composition observed among treatments.

Somatic growth (ANOVA, F_2,6_ = 0.32, *p* = 0.80) and vertebral precipitation rates (ANOVA, F_2,6_ = 1.86, *p* = 0.23; Figure 6d) did not differ among Ba treatments. Mean somatic growth rates ranged from 3.3–6.4 mm DW month^-1^ (Figure 6c) and were consistently and significantly elevated in one tank within each treatment (ANOVA, F ≥ 9.4, *p* ≤ 0.0006 for all comparisons). Tanks with elevated mean growth rates in each treatment represented those that had experienced the least amount of temperature change (1°C) between the temperature and Ba manipulation experiments. Regression analyses indicated D_Zn_ was negatively correlated with somatic growth in two of the three Ba treatments (3x, 6x; Table S2). However, there was no detectable influence of vertebral precipitation rate on D_Zn_ or the other D_Me_ considered in this study (Table S1, S2).

### Classification

The multi-elemental composition of vertebrae successfully distinguished *U. halleri* based on their environmental (i.e. treatment) history (Table 6). Zinc was excluded from DFA because of the observed potential to vary with growth rate. Therefore, DFAs were conducted using four elemental ratios (Mg/Ca, Mn/Ca, Sr/Ca, and Ba/Ca). For the temperature experiment (15°, 18°, 24°C), overall group classification success was 85%, which was significantly better than expected by chance. Classification of rays based on ambient Ba history (1x, 3x, 6x average local values), was accomplished with 96% success overall, which was also better than random chance. Ba/Ca ratios were the most influential variable used to predict group membership in both DFAs.

**Table 6:** Group classification success of round rays (*Urobatis halleri*) determined from discriminant function analysis of vertebral elemental composition of magnesium, manganese, strontium, and barium (expressed as element to calcium ratios).

**Treatment**	**% Correctly Classified**	**Overall % Classification Success**	**κ (± ASE)**
Temperature			
15	91	85	0.77 (0.09)
18	86		
24	76		
Barium			
1x	100	96	0.94 (0.08)
3x	97		
6x	91		

Jack-knife classification success among groups based on known, controlled temperature and dissolved barium concentration histories. Overall percent classification success of groups and chance-corrected classification (κ) ± approximate standard error (ASE) represent independent measures of group classification performance.

## Discussion

We demonstrated that the composition of certain minor and trace metal elements in *U. halleri* vertebrae was related to the physical and chemical properties of the water occupied by the rays. Vertebral incorporation of three of the six elements evaluated demonstrated significant temperature-dependent responses, revealing both positive (D_Mn_) and negative relationships (D_Mg_ and D_Ba_) with temperature. Vertebral Ba/Ca ratios in *U. halleri* were incorporated in proportion to Ba/Ca_water_, supporting their application as useful intrinsic elemental markers. Elemental incorporation of Li, Mg, Mn, Sr, and Ba did not appear to be mediated by somatic growth or vertebral precipitation rates, indicating that individual variation in growth rates are unlikely to be responsible for observed variation in vertebral elemental composition. Significant relationships between somatic growth rate and Zn incorporation were identified. However, correlations between D_Zn_ and somatic growth rate were inconsistent across treatments and between experiments, warranting further investigation. Using DFA, we reliably distinguished the environmental history of individual rays based on differences in vertebral elemental composition. These results indicate that vertebral elemental analysis is a promising tool for the study of elasmobranch populations.

### Vertebral elemental composition and influences on incorporation

#### Lithium

The Li/Ca composition of *U. halleri* vertebrae was occasionally below detection limits, highly variable, and not affected by temperature. In synthetic (nonbiogenic) hydroxyapatite, Li has been found to directly substitute for Ca and to increase proportionately with ambient dissolved concentrations [53]. The sparse experimental work on temperature effects on Li uptake in biogenic calcified structures provides mixed results. Negative temperature effects of Li/Ca incorporation into calcite and aragonite have been identified experimentally in some foraminifera, brachiopods, and coral [54,55] whereas no effect has been observed in other foraminifera and coral species [56].

Otolith Li/Ca has been used as an elemental marker in freshwater [57], diadromous [58], and marine teleosts [59] as well as an elasmobranch [31]. Fleishman et al. [60] determined that lithium (Li^+^) concentrations in the blood plasma of elasmobranchs were 5-7 times lower than that of ambient seawater. This marked discrimination against Li^+^ likely reflects the approach elasmobranchs evolved to maintain internal ionic and osmotic equilibrium (osmoregulation). Because concentrations of NaCl in the plasma of marine elasmobranchs are generally maintained below that of seawater, elasmobranchs experience an osmotic influx of NaCl that must be regulated [60]. Lithium, like Na^+^, is a monovalent alkali metal that is unlikely to be differentiated from Na^+^ during osmoregulation [60]. In elasmobranchs, excess Na^+^, chloride (Cl^‾^) and Li^+^ are concentrated in the kidneys and renal gland and excreted with urine and other waste [61]. Discrimination of Li is reflected in the partition coefficients calculated from *U. halleri* vertebrae in this study (overall D_Li_: 0.85 ± 0.21 SD). However, elemental partitioning may be underestimated in our analyses because of the exclusion of samples that fell below instrument detection limits. If we include those samples as 0 values, the grand mean decreases (D_Li_: 0.64 ± 0.39 SD), indicating a greater extent of Li discrimination.

#### Magnesium

The negative effects of temperature on Mg/Ca incorporation (and D_Mg_) observed in *U. halleri* have also been reported in marine gastropods [62] and benthic foraminifera [63]. Among marine fishes, significant effects of temperature on otolith Mg/Ca ratios have not been commonly reported. Experimental investigations of elemental incorporation into the otoliths of red drum (*Sciaenops ocellatus* [64]), spot (*Leiostomus xanthurus* [16]), gray snapper (*Lutjanus griseus* [65]), and Pacific cod (*Gadus macrocephalus* [42]) all concluded that otolith Mg/Ca was not affected by temperature. Increasing temperature has been found to cause an increase in Mg/Ca within the otoliths of *Argyrosomus japonicus* [66]. In contrast, Mg/Ca ratios in synthetic aragonite show an inverse relationship with temperature similar to that observed within *U. halleri* vertebrae [67]. These results are likely due to underlying differences in the kinetics of mineralization associated with biogenic aragonite and hydroxyapatite and differences in ionic regulation between teleost fishes and elasmobranchs.

Magnesium partition coefficients were expressed across a narrow range (D_Mg_ = 0.32-0.43) and exhibited the strongest discrimination (lowest D_Me_) among the six elements considered in this investigation (e.g. mean D_Mg_ = 0.38, 0.39, 0.39 for 1x, 3x, 6x Ba treatments at 19°C; %CV = 5.6). Magnesium is an essential micronutrient that supports cellular metabolism, immune system function, and skeletal growth, among other physiological processes. In synthetic hydroxyapatites, Mg ions have been found to substitute for and compete with Ca and inhibit mineralization rates [68,69]. Therefore, internal concentrations of Mg are likely subjected to a high degree of physiological regulation that would be reflected in a comparatively consistent pattern of incorporation, as was observed in *U. halleri*. Despite this regulation, Mg may be a useful indicator temperature history and a valuable geospatial marker in elasmobranchs.

#### Manganese

Temperature influences on Mn incorporation into biomineralized structures have generally been found to be insignificant [65,70] or negative [40,71], but Mn/Ca_vertebrae_ and D_Mn_ were positively affected by temperature in *U. halleri*. The effect was not expressed across temperatures but was driven by significantly lower incorporation of Mn/Ca within the coldest (15°C) treatment. Manganese is an essential micronutrient that is an important cofactor for many enzymes and supports metabolism, protein production, cellular signaling processes, and the activation of reproductive hormones. Though Mn/Ca uptake is proportional to Mn/Ca_water_ in synthetic hydroxyapatite [72], osmotic regulation of Mn ions would alter this direct relationship in biogenic hydroxyapatites. Furthermore, diet represents the primary pathway of Mn uptake in elasmobranchs and other vertebrates [73,74]. In a comparative study of radionuclide accumulation in a teleost and elasmobranch, Pentreath [75] concluded that uptake of Mn radioisotopes solely from water was insufficient to explain internal concentrations of the radionuclide. More recently, Mathews & Fisher [74] experimentally determined that >90% of the Mn accumulated in the soft tissues of lesser spotted dogfish (*Scyliorhinus canicula*) was derived from dietary sources. The contribution of dietary Mn in addition to uptake from the environment at the gills offers an explanation for the elevated values of Mn/Ca_vertebrae_ in comparison to water Mn/Ca in our experiment (Figure 2c).

Considerable variation in D_Mn_ has been reported within and among species. D_Mn_ ranged between 0.10–1.90 in juvenile black bream (*Acanthopagrus butcheri* [76]), 0.018–1.02 in grey snapper [65], and 7.67–32.83 in a field-based study of spotted sea trout (*Cynoscion nebulosus* [77]). Strasser et al. [78] identified ontogenetic differences in D_Mn_ between larval (mean ± SD: 1.86 ± 0.19) and juvenile softshell clams (mean ± SD: 0.88 ± 0.13), *Mya Arenaria*. Our estimates of D_Mn_ typically exceeded 1.0 (temperature experiment: 0.9–1.60; Ba manipulation experiment: 1.36–1.69) but fell within the broad range reported among these other investigations. Laboratory studies intended to assess the factors controlling Mn incorporation into otoliths have found no evidence of a relationship between Mn/Ca_water_ and Mn/Ca_otolith_ (see review by Miller [40]). However, Limburg et al. [79] hypothesized that cyclical variation of Mn/Ca_otolith_ ratios were associated with migrations into deep water hypoxic zones that are characterized by elevated Mn concentrations, providing historic records of hypoxia intensity. Further research on the mechanisms of Mn incorporation is needed to clarify the utility of this element as a geospatial tag or indicator environmental history.

#### Zinc

Our analyses of Zn incorporation into *U. halleri* vertebrae revealed a positive influence of temperature on Zn/Ca_vertebrae_, no significant effect of temperature on D_Zn_, and significant influences of somatic growth rates on D_Zn_, providing a somewhat convoluted perspective on the factors influencing Zn incorporation. Few studies have attempted to experimentally validate Zn incorporation into biogenic calcified structures [70,71,80]. Zn is fundamental to a diverse array of physiological processes, including growth, neurotransmission, and cell signaling. It plays a vital role in protein production, structure, and maintenance [81]. Though branchial uptake of Zn is not inconsequential, diet represents the primary source of Zn intake in both elasmobranch and teleost fishes [74,75]. The dietary contribution of Zn in the elasmobranch *S. canicula* (>80% [74]) is similar to those experimentally estimated for other fishes [82,83]. Given our use of standardized diets, the observed inconsistencies may be the result of the variation in Zn/Ca_water_ values (Tables 1, 4). Alternatively, variation in Zn/Ca_vertebrae_ may be influenced by somatic growth rate and kinetic effects.

In synthetic hydroxyapatites, Zn substitution for Ca is minimal and the majority of Zn is incorporated through inclusion into interstitial spaces [84]. Elements incorporated into interstitial spaces can be representative of environmental conditions [8,15], but the pathways and mechanisms of Zn incorporation may differ in biogenic hydroxyapatite. Miller et al. [85] determined that the majority (40-60%) of Zn contained in Atlantic cod (*Gadus morhua*) otoliths was associated with the protein matrix rather than the mineralized aragonite structure or interstitial spaces. Given the critical role of Zn identified in more than 300 fish proteins [13], it is likely that much of the Zn contained in the biogenic hydroxyapatite of elasmobranch vertebrae is bound within the protein matrix as well. Because it is prevalent in the protein structure of otoliths and is assimilated primarily through dietary sources, Miller et al. [85] concluded that Zn is unlikely to be a reliable proxy of ambient environmental conditions. Zn has been reported to be useful for distinguishing shark populations [30] and movements of sharks between habitats [31]. Based on our results and a review of available literature, we do not anticipate vertebral Zn/Ca ratios and D_Zn_ to be commonly representative of ambient conditions.

#### Strontium

Strontium is one of the most commonly studied elemental markers in biogenic calcified structures. Unlike several of the elements previously considered in this study, a physiological role for Sr has not been identified in fishes [14]. Sr is primarily derived via branchial uptake in fishes and Sr/Ca ratios of otoliths are typically representative of ambient concentrations [86–88]. In synthetic hydroxyapatite and aragonite, Sr is known to compete with and substitute for Ca [89]. Temperature-dependent responses in Sr/Ca incorporation have provided reliable indicators of temperature history in corals [90] and fishes [39], but Sr incorporation was not influenced by temperature in *U. halleri*. Temperature-independent patterns of Sr/Ca incorporation were also reported in juvenile *L. xanthurus* scales [91], common cuttlefish statoliths (*Sepia officinalis* [38]), and the otoliths of European eels (*Anguilla anguilla* [92]). We anticipate that Sr is likely to be a valuable elemental marker in elasmobranchs, particularly among euryhaline species.

#### Barium

The strong, negative effect of temperature on Ba incorporation in *U. halleri* is similar to the pattern observed by Balter & Lécuyer [93] in laboratory studies of synthetic hydroxyapatite. Decreases in Ba/Ca ratios with increasing ambient water temperature have also been found in laboratory studies with synthetic aragonite [67], cephalopods [38], larval gastropods [91,94], juvenile clams [95], and larval fish [42]. However, positive or no effect of temperature on Ba incorporation into otoliths has been much more commonly observed [40]. Studies of Ba incorporation into the hydroxyapatite of fish scales, bone, and teeth have also revealed either positive [12] or no relationship to temperature [91]. Given the inconsistency in temperature effects on Ba incorporation reported within the literature, it is likely that species-specific variation in this temperature response is widespread.

The significant positive relationship between vertebrae and water Ba/Ca supports the utility of Ba/Ca ratios as elemental markers. However, our observation of a negative temperature effect on Ba/Ca incorporation indicates there are likely to be interactive effects of temperature and water concentration on vertebral Ba incorporation. These effects could confound interpretations of field data, particularly in study areas with sharp gradients in both temperature and Ba/Ca_water_. For example, vertebral Ba/Ca of *U. halleri* at 15.4°C and exposed to Ba/Ca_water_ of 6.3 μmol mol^-1^ (Ba/Ca_vertebrae_ = 0.96) would be indistinguishable from *U. halleri* that had resided in water averaging 19.7°C with a mean Ba/Ca_water_ concentration of 31.7 μmol mol^-1^ (Ba/Ca_vertebrae_ = 0.94). This finding highlights the importance of experimental validation studies, the utility of measuring multiple elemental markers, the value of temperature data from study areas, and need for caution when interpreting patterns among elemental signatures from field studies.

### Effects of growth and precipitation rates

Variation in growth rates can alter physiological and kinetic processes that directly modify patterns and rates of elemental discrimination and incorporation [47,71]. For example, growth-mediated effects on elemental incorporation could produce significant variability among individuals with inherently different growth rates that occupy the same water mass. Indeed, growth rates have been found to influence the composition of some calcified structures [7,16,18]. In this study, we found no significant relationships between somatic growth or vertebral precipitation rates and D_Me_ for any elements except Zn in *U. halleri* (Table S1, S2), which supports the premise that growth rates do not generally alter vertebral elemental composition.

In contrast to other elements, D_Zn_, independent of temperature, was significantly but inconsistently correlated with somatic growth. The effect of growth on D_Zn_ was restricted to the 3x and 6x Ba treatments (Table S1, Table S2). Vertebral precipitation rates (μm radius month^-1^), however, were not significantly correlated with D_Zn_. Zn/Ca_water_ values displayed the greatest variance among the six elemental ratios measured in this study (%CV = 47.8). Dissolved Zn concentrations can be highly variable, elevating during periods of increased river discharge and runoff [96]. Water changes that occurred during high flow events could have influenced Zn/Ca_water_ and Zn/Ca_vertebrae_ in some tanks. Trace levels of Zn in seawater are also prone to contamination [97]. Given broad environmental variation and the potential for contamination, our sampling frequency may not have been sufficient to adequately characterize the uptake and partitioning of Zn in *U. halleri*. Further research is needed to clarify the relationships between Zn/Ca_water_, Zn/Ca_vertebrae_, and somatic growth rates, if this element is to be used as a reliable marker of population structure, habitat use or natal origins.

### Ecological applications and future directions

Group classification of *U. halleri* based on environmental history within the controlled laboratory studies was highly successful (Table 6). Our results indicate that elemental variation in elasmobranch vertebrae can reliably distinguish individuals based on differences in their environmental history or habitat – assuming differences among those habitats or time periods exist. Studies of vertebral elemental chemistry could discern natal origins, biological hotspots, movement patterns, habitat use, and population structure of elasmobranchs, and generate critical information for spatially-explicit conservation and management measures.

The significant temperature effects and the likelihood for interaction between temperature and ambient concentration on Ba incorporation observed in this study emphasize the importance of considering multiple elemental markers when making spatial and temporal inferences regarding environmental history. Measurements of vertebral bulk or compound-specific stable isotopic composition [98,99], mapping of environmental chemical composition/isoscapes [100,101], or molecular analyses [102] used in conjunction with minor and trace elemental assays should provide greater resolution than would be obtained from a single method alone. We anticipate that studies integrating complementary intrinsic markers will generate corroborative and more robust conclusions based on field data.

Our results prompt questions regarding the periodicity of growth band/increment deposition within elasmobranch vertebrae. Increments are deposited daily within fish otoliths and bivalve shells, a phenomenon that has not yet been found in elasmobranchs [4,26]. Yet, microscopic examination of elasmobranch vertebrae typically reveals other increments and checks within the pair of annual growth bands [26,103]. Are growth bands deposited at finer temporal scales within elasmobranch vertebrae? The ability to reconstruct environmental history and assay elemental markers with more refined temporal resolution would enhance the utility of this tool in studies of elasmobranch populations.

Elemental composition of elasmobranch vertebrae may not provide a useful record of environmental history for all species. Vertebral elemental composition could differ due to species-specific environmental tolerances [104,105] or extent of vertebral calcification [24,106]. Additional laboratory or field-based experiments should be pursued to gain insight into the potential differences in elemental incorporation among species. Our validation experiments advance the use of vertebral elemental markers for the study of elasmobranch populations and provide a framework for interpreting the results of future field investigations.

## Supporting Information

Table S1 **Correlations (r) between partition coefficients (D_Me_) and somatic growth rates and vertebral precipitation rates for the temperature (T) experiment.** The number (n) of round rays (*Urobatis halleri*) included in growth rate estimates, observed range of individual somatic growth rates (mm disc width month^-1^), and vertebral precipitation rates (μm radius month^-1^) are reported for each treatment. No significant correlations were detected (p ≥ 0.12).
(PDF)

Table S2 **Correlations (r) between partition coefficients (D_Me_) and somatic growth rates and vertebral precipitation rates for the barium manipulation ([Ba]) experiment.** The number (n) of round rays (*Urobatis halleri*) included in growth rate estimates, observed range of individual somatic growth rates (mm disc width month^-1^), and vertebral precipitation rates (μm radius month^-1^) are reported for each treatment. Significant p-values are indicated by bold font.
(PDF)
