# Supplementary material for: Elemental Markers in Elasmobranchs: Effects of Environmental History and Growth on Vertebral Chemistry
Source: PLoS One. 2013 Oct 1;8(10):e62423. doi: 10.1371/journal.pone.0062423 (PMC3787939; doi:10.1371/journal.pone.0062423)
Supplement: Table S1 — Correlations (r) between partition coefficients (DMe) and somatic growth rates and vertebral precipitation rates for the temperature (T) experiment. The number (n) of round rays (Urobatis halleri) included in growth rate estimates, observed range of individual somatic growth rates (mm disc width month-1), and vertebral precipitation rates (μm radius month-1) are reported for each treatment. No significant correlations were detected (p ≥ 0.12). (PDF) [file pone.0062423.s001.pdf]

Table S1. Supporting information. Correlations (*r*) between partition coefficients (D_{Me}) and somatic growth rates and vertebral precipitation rates for the temperature (T) experiment. The number (n) of round rays (*Urobatis halleri*) included in growth rate estimates, observed range of individual somatic growth rates (mm disc width $month^{-1}$), and vertebral precipitation rates (µm radius $month^{-1}$) are reported for each treatment. No significant correlations were detected ($p \geq 0.12$).

		Somatic growth rate				Precipitation rate	
**Treatment**	**D_{Me}**	***r***	***p***	**Treatment**	**D_{Me}**	***r***	***p***
T = 15 °C	Li	0.288	0.162	T = 15 °C	Li	0.041	0.970
n = 32	Mg	0.035	0.849	n = 28	Mg	0.408	0.213
Range: 0.5-3.3 mm $month^{-1}$	Mn	0.269	0.174	Range: 5.5-31.2 µm $month^{-1}$	Mn	0.017	0.960
	Zn	0.119	0.545		Zn	0.323	0.333
	Sr	0.296	0.127		Sr	0.297	0.324
	Ba	0.051	0.779		Ba	0.275	0.388
T = 18 °C	Li	0.289	0.181	T = 18 °C	Li	0.219	0.472
n = 33	Mg	0.298	0.103	n = 30	Mg	0.193	0.473
Range: 1.4-4.5 mm $month^{-1}$	Mn	0.173	0.369	Range: 12.8-49.6 µm $month^{-1}$	Mn	0.245	0.379
	Zn	0.280	0.128		Zn	0.320	0.226
	Sr	0.184	0.323		Sr	0.337	0.202
	Ba	0.029	0.889		Ba	0.283	0.307
T = 24 °C	Li	0.248	0.215	T = 24 °C	Li	0.023	0.919
n = 33	Mg	0.208	0.246	n = 30	Mg	0.117	0.552
Range: 3.6-8.0 mm $month^{-1}$	Mn	0.202	0.272	Range: 36.7-101.0 µm $month^{-1}$	Mn	0.037	0.855
	Zn	0.139	0.465		Zn	0.318	0.121
	Sr	0.193	0.282		Sr	0.089	0.653
	Ba	0.264	0.166		Ba	0.109	0.603
