# Supplementary material for: Elemental Markers in Elasmobranchs: Effects of Environmental History and Growth on Vertebral Chemistry
Source: PLoS One. 2013 Oct 1;8(10):e62423. doi: 10.1371/journal.pone.0062423 (PMC3787939; doi:10.1371/journal.pone.0062423)
Supplement: Table S2 — Correlations (r) between partition coefficients (DMe) and somatic growth rates and vertebral precipitation rates for the barium manipulation ([Ba]) experiment. The number (n) of round rays (Urobatis halleri) included in growth rate estimates, observed range of individual somatic growth rates (mm disc width month-1), and vertebral precipitation rates (μm radius month-1) are reported for each treatment. Significant p-values are indicated by bold font. (PDF) [file pone.0062423.s002.pdf]

Table S2. Supporting information. Correlations (*r*) between partition coefficients (D_{Me}) and somatic growth rates and vertebral precipitation rates for the barium manipulation ([Ba]) experiment. The number (n) of round rays (*Urobatis halleri*) included in growth rate estimates, observed range of individual somatic growth rates (mm disc width month^{-1}), and vertebral precipitation rates (µm radius month^{-1}) are reported for each treatment. Significant p-values are indicated by bold font.

		Somatic growth rate				Precipitation rate	
**Treatment**	D_{Me}	***r***	***p***	**Treatment**	D_{Me}	***r***	***p***
[Ba] = 1x	Li	0.163	0.479	[Ba] = 1x	Li	0.421	0.105
n = 34	Mg	0.089	0.628	n = 30	Mg	0.006	0.979
Range: 1.8-7.8 mm month^{-1}	Mn	0.210	0.265	Range: 7.3-80.1 µm month^{-1}	Mn	0.258	0.259
	Zn	0.327	0.083		Zn	0.043	0.854
	Sr	0.175	0.338		Sr	0.076	0.723
	Ba	0.012	0.953		Ba	0.337	0.146
[Ba] = 3x	Li	0.101	0.622	[Ba] = 3x	Li	0.172	0.525
n = 34	Mg	0.003	0.960	n = 29	Mg	0.167	0.471
Range: 2.0-6.5 mm month^{-1}	Mn	0.209	0.260	Range: 8.9-79.6 µm month^{-1}	Mn	0.016	0.949
	Zn	0.462	**0.013**		Zn	0.103	0.684
	Sr	0.176	0.320		Sr	0.157	0.496
	Ba	0.087	0.635		Ba	0.050	0.835
[Ba] = 6x	Li	0.022	0.932	[Ba] = 6x	Li	0.039	0.900
n = 29	Mg	0.197	0.297	n = 28	Mg	0.173	0.454
Range: 2.0-7.3 mm month^{-1}	Mn	0.126	0.522	Range: 7.3-73.4 µm month^{-1}	Mn	0.228	0.334
	Zn	0.498	**0.010**		Zn	0.097	0.296
	Sr	0.086	0.665		Sr	0.253	0.296
	Ba	0.244	0.229		Ba	0.428	0.076
